# Nef-M1, a CXCR4 Peptide Antagonist, Enhances Apoptosis and Inhibits Primary Tumor Growth and Metastasis in Breast Cancer

**DOI:** 10.4236/jct.2013.44101

**Published:** 2013-06-01

**Authors:** Harvey Bumpers, Ming-Bo Huang, Venkat Katkoori, Upender Manne, Vincent Bond

**Affiliations:** 1 Department of Surgery, Michigan State University College of Human Medicine, Lansing, USA; 2 Department of Biochemistry Microbiology and Immunology, Morehouse School of Medicine, Atlanta, USA; 3 Department of Pathology, University of Alabama at Birmingham, Birmingham, USA

**Keywords:** Breast Cancer, Nef-M1, CXCR4, Apoptosis, Metastasis

## Abstract

Results from studies with animal models suggest that, in many cancers, CXCR4 is an important therapeutic target and that CXCR4 antagonists may be promising treatments for primary cancers and for metastases. The Nef protein effectively competes with CXCR4’s natural ligand, SDF-1α, and induces apoptosis. As described in this report, the Nef-M1 peptide (Nef protein amino acids 50 – 60) inhibits primary tumor growth and metastasis of breast cancer (BC). Four BC cell lines (MDA-MB-231, MDA-MB-468, MCF 7, and DU4475) and primary human mammary epithelium (HME) cells were evaluated for their response to the Nef protein and to the Nef-M1 peptide. The presence of CXCR4 receptors in these cells was determined by RT-PCR, Western blot (WB), and immunohistochemical analyses. The apoptotic effect of Nef-M1 was assessed by terminal transferase dUTP nick-end labeling (TUNEL). WBs was used to assess caspase 3 activation. BC xenografts grown in SCID mice were evaluated for the presence of CXCR4 and for their metastatic potential. CXCR4 was presented in MDA-MB-231, MCF 7, and DU 4475 BC cells but not in MDA-MB-468 BC or HME cells. Cells expressing CXCR4 and treated with Nef-M1 peptide or the Nef protein had higher rates of apoptosis than untreated cells. Caspase-3 activation increased in MDA-MB 231 cells treated with the Nef protein, the Nef 41 – 60 peptide, or Nef-M1. Nef-M1, administered to mice starting at the time of xenograft implantation, inhibited growth of primary tumors and metastatic spread. Untreated mice developed diffuse intraperitoneal metastases. We conclude that, in BCs, Nef-M1, through interaction with CXCR4, inhibits primary tumor growth and metastasis by causing apoptosis.

## 1. Introduction

In American women, breast cancer (BC) is the most frequent cancer and the second leading cause of cancer-related deaths. In 2013, 232,340 new cases of invasive BC are estimated to occur among women, and approximately 39,620 women are expected to die from BC [1]. Since the etiology of BC is unknown, attempts to eliminate this disease are based on early detection and treatment. Although many risk factors have been identified [2], none showed a direct link to its etiology, which appeared to be multifactorial. The ability to cure BC patients progressively decreases as stages of the disease advance, but some patients can be cured through all stages, except Stage IV (distant metastasis). In Stage IV disease, the focus shifts to increase the length of survival by use of systemic therapy to slow tumor growth and inhibit metastasis [3]. Patients succumb to the disease when metastases have invaded vital organs (liver, lung, brain), and the tumor’s growth rate can no longer be controlled. Therefore, identification of the molecules that promote metastasis is necessary to produce targeted therapy.

Certain chemokines and their receptors, in particular stromal cell-derived factor (SDF)-1α and CXC chemokine receptor 4 (CXCR4), are involved in cancer cell migration, proliferation, and survival [4]. SDF-1α and its unique receptor, CXCR4, are expressed in various epithelial cancer cells and are associated with tumorigenesis [5–7]. Over-expression of CXCR4 is associated with lymph node metastasis and BC stages III/IV and reduced survival of patients with gastric cancer [8].

CXCR4 is highly expressed in human BC cells, and activation of the receptor with SDF-1α induces chemotaxis and tissue invasion [9]. Further, inhibition of the interaction of SDF-1α and CXCR4 impairs metastasis of BC cells to regional lymph nodes and lung, suggesting that chemokines and their receptors are involved in determining the metastatic destination of tumor cells [9].

The Nef gene of human immunodeficiency virus (HIV) encodes a 27 – 34 kD myristoylated protein, which is expressed early after establishment of the provirus in host cells. Nef protein competes with CXCR4’s natural ligand, SDF-1α, and induces apoptosis [10]. The apoptotic motif in the HIV-1 Nef protein (Nef-M1) is cytotoxic to various cultured human cancer cell lines, including BC. The Nef-M1 peptide is an apoptotic activator and inhibitor of growth and metastasis of primary colorectal cancer (CRC) cells [11,12]. In previously reported work [10], we identified the apoptotic motif in the Nef protein and determined its effects on cell cultures. In this report, the focus is on its role of inhibiting tumor growth and metastasis of human BC.

## 2. Materials and Methods

### 2.1. Peptides and Antibodies

Nef-Motif-1 (Nef-M1 or M1) and Nef sMotif-1 (Nef-sM1 or sM1, the scrambled amino acid sequence of Nef-M1) were obtained from Sigma Genosys (Houston, TX). Antibodies used includes monoclonal mouse anti-human fusin clone 12G5, mIgG2a (CXCR4) (Research and Diagnostics Inc., Flanders, NJ); anti-mouse flourescein isothiocynate, mIgG (H + L) made in goat (Pierce Biotechnology, Rockford IL).

### 2.2. Cell Cultures

Four human BC cell lines (MDA-MB231, MDA-MB468, MCF7, and DU4475) and one normal mammary epithelial cell line (HME) were used. Each cell line was originally purchased from American Type Culture Collection (ATCC, Manassas, VA) and cryopreserved. All cell lines were cultured in 5% CO_2_ at 37°C in RPMI 1640 medium (Invitrogen, Palo Alto, CA.) supplemented with L-glutamine (2 mM) (Cellgro, Fisher Scientific, Suwanee, GA), 10% fetal bovine serum (Biowhittaker-Cambrex, Walkersville, MD), and penicillin (100 U/mL)/streptomycin (100 U/mL) (Biowhittaker-Lonza). Cell cultures were grown to 80% confluence and injected into mice according to an established protocol.

### 2.3. Total RNA Isolation and Reverse Transcription (RT)-PCR

Total RNA was extracted from MDA-MB231 and MDA-MB468 samples using the RNAzolTM B (TEL-TEST, Inc., Friendswood, TX) following the manufacturer’s instructions. mRNA expression of CXCR4 was determined by RT-PCR. Total RNA (5 µg) was reverse transcribed into cDNA with SuperScriptTM III One-Step RT-PCR System with Platinum Taq DNA Polymerase Kits (Invitrogen Life Technologies, Carlsbad, CA). The reaction was accomplished in 50 µL mixtures maintained at 55°C for 30 min, followed immediately by denaturing at 94°C for 2 min. The following sequences of human CXCR4 primers used for PCR were: a) hCXCR4-1, 1097 bp of CXCR4 (forward): 5’-atgaaacttggggcgaggac-3’; (reverse): 5’-cggtgtagttatctgaagtg-3’; b) hCXCR4-2, 922 bp of CXCR4 (forward): 5’-atgtccattcctttgcctct-3’; (reverse): 5’-aaagcatagaggatggggtt-3’; and c) hCXCR4-3, 508bp of CXCR4 (forward): 5’-tacctggccatcgtccacgc-3’; (reverse): 5’-tccaaacacgagtgcatacc-3’. cDNA synthesis and denaturation were accomplished at 55°C for 30 min and at 94°C for 2 min, then 35 cycles of PCR amplification included denaturation at 94°C for 15 sec, annealing at 60°C for 30 sec, extended at 68°C for 1 min, and final extension at 68°C for 5 min. Region 2984 to 4081 of human CXCR4 were amplified (GenBank accession no. AF005058). PCR products were visualized on 1.5% agarose gels containing ethidium bromide and analyzed with Fotodyne FOTO/Analyst Luminary Workstations (Fotodyne, Inc., Hartland, WI). The PCR products were purified by use of a QIAquick Gel Extraction Kit (QIAGEN, Valencia, CA) and sequenced by ABI Applied Biosystems, 3130 x1, Genetic Analyzer Data Collection software V3.0 (ABI, Foster City, CA).

### 2.4. Western Blot Analysis for CXCR-4

Cells were grown to 80% confluence in 6 well plates for 48 hr. Cells were washed twice in ice-cold PBS containing 1 mM Na_3_VO_4_, and incubated at room temperature (RT) for 2 min in 200 µL of lysis solution (1.0% Nonidet P-40 [NP-40; Sigma]; 50 mM Tris-HCl, pH 7.5; 20 mM EDTA buffer). The lysates were centrifuged for 20 min at 12,000 rpm at 4°C. The supernatants were collected and stored at −70°C. Protein concentrations were determined with the Bradford assay kit (Bio-Rad Laboratories, Hercules, CA). Portions of each sample (25 µl) were separated by SDS-PAGE on a 4% – 20% Tris-HCl Criterion precast gel (Bio-Rad Laboratories) and electrophoretically transferred to nitrocellulose membranes. The membranes were washed in 1× Tris-buffered saline (TBS) for 5 min, and then blocked with 5% nonfat milk in 1× TTBS (1× TBS and 0.1% Tween 20) for 1 hr by shaking at RT. For detection of CXCR4 protein expression, a mouse anti-human CXCR4-specific antibody was used. This was accomplished by shaking the membranes at 4°C overnight, as directed by the manufacturer, followed by application of horseradish peroxidase (HRP)-conjugated goat anti-mouse antibody (H + L). Protein bands were detected by Western Blotting Luminol Reagent. After detection of CXCR4, the blots were stripped and hybridized with a monoclonal mouse anti-α-tubulin (clone B-5-1-2), then probed with the HRP-conjugated goat anti-mouse antibody (H + L).

### 2.5. Immunocytochemistry

MDA-MB231 and MDA-MB468 cells were grown in RPMI1640 medium with 10% fetal bovine serum in 35-mm plates for 48 hr. Cells were rinsed with PBS containing 0.1% glycine to reduce intrinsic fluorescence and blocked with 1% goat serum in PBS containing 0.3% Triton X-100 at RT for 1 hr. The cells were stained with an anti-CXCR4 primary antibody (1:250) at 4°C overnight. The plates were rinsed with PBS containing 1% Triton X-100 at RT, exposed to a secondary antibody tagged with fluorescent isothiocyanate (FITC), and washed again with PBS. Images were taken by fluorescence microscopy (magnification, ×400) and arranged with Adobe Photoshop 5.0.2 software.

### 2.6. Nef-M1 Peptide Dose Response

Dilution of the Nef-M1 peptide or protein was accomplished according to a previously reported protocol [11, 12]. Dose responses were assessed by incubating 2.5 × 10^5^ MDA-MB231, MDA-MB468, MCF7, or HME cells with the Nef-M1 peptide or the intact Nef protein at various concentrations in 35-mm multiwall plates for 24 hr. The concentrations of Nef-M1 peptide or Nef protein were 0, 0.01, 0.1, 1, 10, and 100ng/mL. Analysis was by terminal deoxynucleotidyl transferase dUTP nick-end labeling (TUNEL).

### 2.7. TUNEL Assay

To evaluate apoptosis, TUNEL assays were performed with an *in situ* cell death detection protocol. The procedure for immunohistochemical detection and quantification of apoptosis was based on labeling of DNA breaks. The cells were treated with Nef-M1peptide or Nef protein at 37°C for 24 hr. Cells were washed with PBS, then fixed with 1 mL of a freshly prepared solution of 4% paraformaldehyde in PBS, pH 7.4, for 1 hr at RT. Cells were rinsed with PBS and incubated in permeabilization solution (0.1% Triton X-100, 0.1 sodium citrate) for 10 min at RT. The cells were rinsed with PBS, and 50 µL of TUNEL reaction mixture, consisting of TdT and biotinylated nucleotides was added. The cells were incubated in a humidified chamber for 1 hr at 37°C and rinsed three times with PBS. Samples were analyzed under a fluorescence microscope. The values derived were a compilation of at least three independent experiments, and bars were used to show the standard errors of the measurements.

### 2.8. Caspase-3 Analysis

After being treated with scrambled Nef-sM1 (Nef-sM1), Nef-171–180, Nef protein, Nef 41 – 60, or Nef-M1peptide, MDA-MB231 cells cultured in 6 well plates were harvested. WB analysis and a monoclonal mouse anti-caspase-3 antibody (Active Motif Inc, Carlsbad, and CA) were used to characterize the expression of caspase-3. A monoclonal mouse anti-α-tubulin (clone B-5-1-2) (Sigma) was used to detect expression of α-tubulin for a loading control. Caspase-3 protein bands were detected by Western Blotting Luminol Reagent, followed by exposure to photographic BioMax film (Fisher Scientific, Pittsburgh, PA). Images were scanned into Adobe Photoshop 5.0.2, and densitometry was performed using Scion Imaging software, Release Beta 3b (Scion Corporation, Frederick, MD).

### 2.9. Animals

Severe combined immunodeficient (SCID) female mice were purchased from Taconic Farms (Taconic, NY) at four weeks of age and quarantined for one week prior to use. The mice were inoculated with BC cells or tumor tissue implants to establish primary tumors or to metastasize to the liver. Food, water, and bedding were sterilized by autoclaving. The mice were kept in micro-filtered cages in a room designated for immune-compromised mice. On a daily basis, the animals were evaluated regarding their health status and tumor growth. Body weights, nutritional intake, general activity, and ruffling of fur were used to determine the health status. All surgical procedures were accomplished under a laminar flow hood and with sterile protocols. A liquid sterilant, Exspor (Alcide Co., Norwalk, CT) was used to sanitize the gloves of handlers and mouse skin at the site of planned surgery.

### 2.10. Tumor Implantation and Nef-M1 Peptide Injections

MDA-MB231 cells (5 × 10^6^ in 0.1 mL of Hanks balanced salt solution) were injected subcutaneously. For primary growth of tumors, the injections were made into the flank. Tissue implants were also performed. A solid tumor developing after injection of cells was cut into 2 – 4 mm pieces in serum-free culture media and kept at 4°C until used. The mice were sedated with 0.6 mL of avertin (2,2,2-tri-bromoethanol and 2-methyl-2-butanol). To assess metastatic potential, tumors were implanted subcutaneously in mouse mammary tissue or in the gonadal fat. Surgical wound closures were made using 5 - 0 absorbable sutures or skin staples. Following tumor implantation, the mice were placed under a heat lamp for 10 min to recover and then placed back in their cages. At 2 hr after the procedure, they were checked for recovery and stability. Starting at the time of tumor implantation, the mice were dosed intraperitoneal (2 micrograms biweekly) with the Nef-M1 peptide or with the vehicle.

## 3. Results

### 3.1. Expression of CXCR4 in BC or Normal Cell Lines

**Figure 1:**
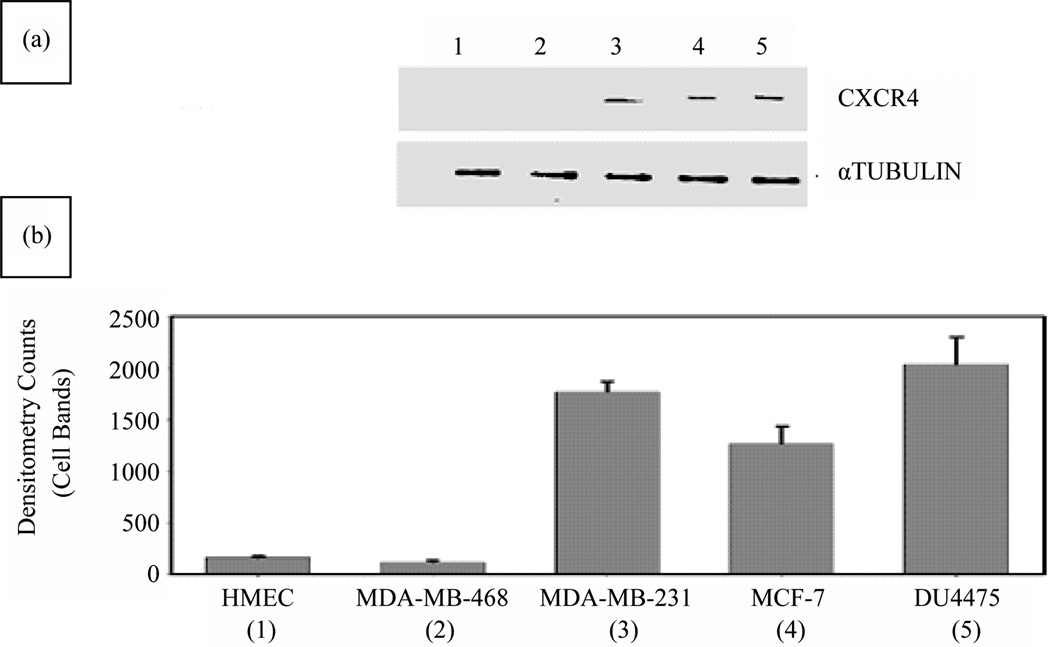
Evaluation of BC and HME cell lines for CXCR4 expression status by WB analysis. (a) The CXCR4 expression status of MDA-MB231, MDA-MB468, MCF7, DU4475, and HME cells was determined. WB analysis confirmed a lack of expression of CXCR4 in HME (lane 1) and MDA-MB468 (lane 2) cells and the presence of CXCR4 in MDA-MB231 (lane 3), MCF7 (lane 4), and DU4475 (lane 5) cells; (b) Densitometry analysis of WBs showed barely detectable expression of CXCR4 in HME (lane 1) and MDA-MB468 (lane 2) cells but higher expression of CXCR4 in MDA-MB231 (lane 3), MCF7 (lane 4), and DU4475 (lane 5) cells.

**Figure 2:**
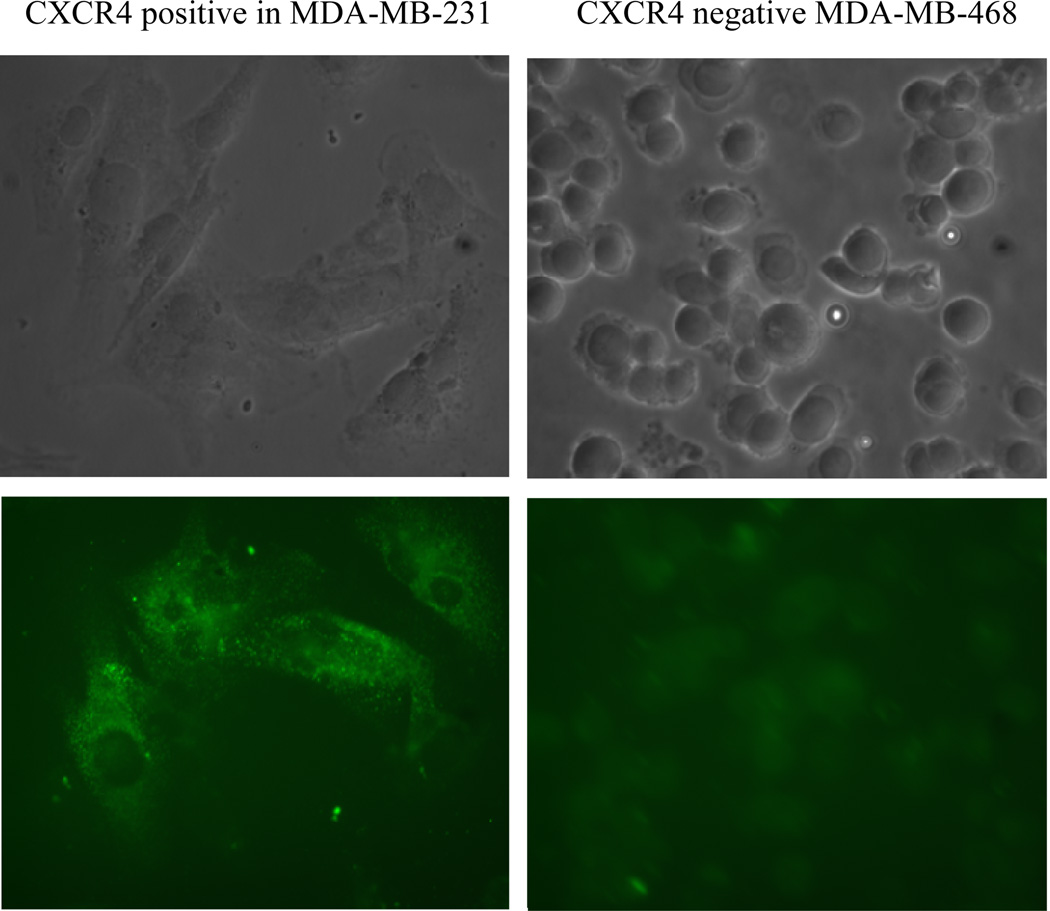
Immunocytochemical evaluation of the expression of CXCR4 in MDA-MB468 and MDA-MB231 cells. The intensity of green fluorescence demonstrated the presence of CXCR4 in MDA-MB231 cells and its absence in MDA-MB468 cells.

As determined by WB analysis, there was CXCR4 phenotypic expression in three BC cell lines, MDA-MB231, MCF 7, and DU 4475; in contrast, there was no expression in MDA-MB468 cells or in HME cells (Figure 1(a)). RT-PCR analysis demonstrated expression of the mRNA for CXCR4 in the same three BC cell lines, Again, MDA-MB468 BC cells and HME cells showed no expression (data not shown). The BC cell lines that were positive for CXCR4 exhibited varying degrees of expression on their surface or in the cytoplasm (Figure 1(b)). The expression status of CXCR4 in MDA-MB231 and MDA-MB468 cells was confirmed by immunocytochemistry. In this analysis, MDA-MB231 cells were strongly positive (bright fluorescent green stain) (Figure 2) for CXCR4 expression. CXCR4 expression was not observed in MDA-MB468 cells.

### 3.2. Effect of Nef-M1 Peptide or the Nef Protein on Apoptosis in BC

**Figure 3:**
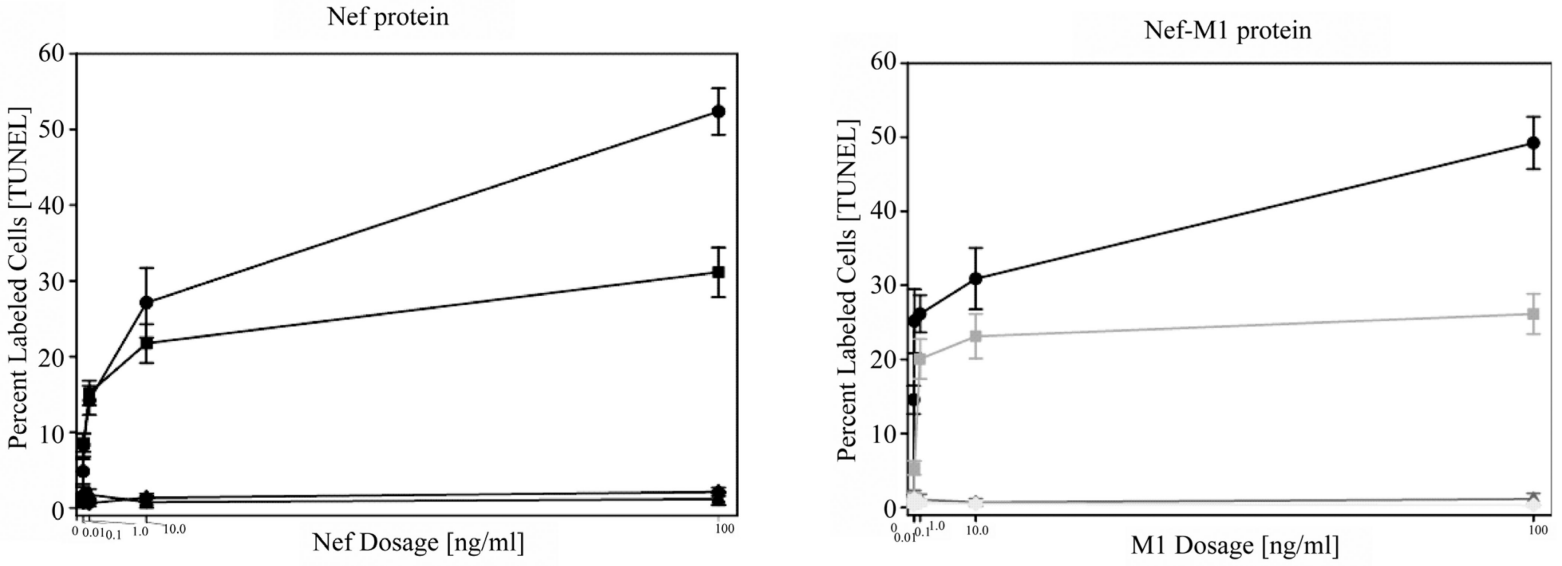
Nef-M1 peptide dose response as determined by TUNEL assays. Dose responses to the full Nef protein and the Nef-M1 peptide were determined at concentrations of 0, 0.01, 0.1, 1, 10, and 100 ng/ml in CXCR4-negative (MDA-MB468, HME) and -positive (MDA-MB231, MCF7) BC cell lines. The time of exposure was 24 hr. For MDA-MB231 and MCF7 cells, the percent of labeled cells increased with increasing concentrations of the Nef-M1 peptide, whereas there was no change in the percent of labeled cells in MDA-MB468 cells and HME cells. Graph symbols for cell lines: MDA MB231 (circle), MCF7 (square), MDA-MB468 (diamond), HME (triangle).

**Figure 4:**
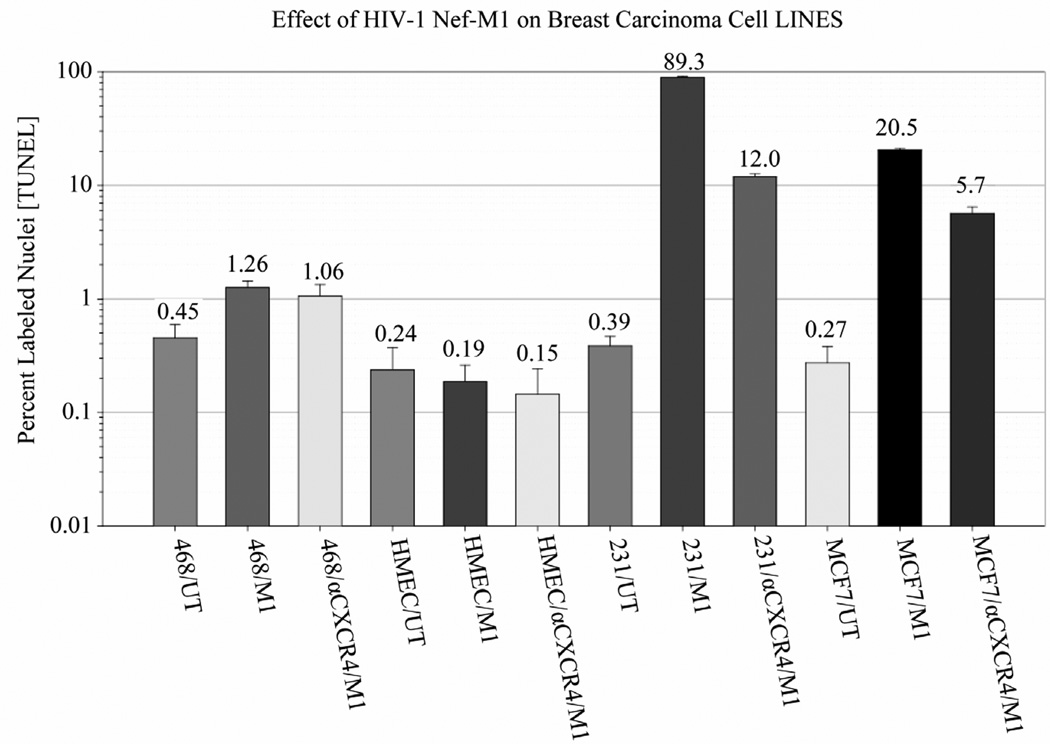
Effect of the Nef-M1 peptide (M1) and anti-CXCR4 linked Nef-M1 mab (αCXCR4/M1) on MDA-MB468, HME, MDA-MB231, and MCF7 cells, as determined by TUNEL assays. Relative to untreated cells expressing CXCR4 receptors, the Nef-M1 peptide was efficient in stimulating apoptosis of cells, as determined after 48 hr of exposure. Furthermore, for cells expressing CXCR4, there were similar levels of apoptosis following treatments with Nef-M1 or αCXCR4/M1.

The effects of the CXCR4 antagonists, Nef-M1 peptide and Nef protein, on apoptosis in BC cell lines was evaluated by TUNEL assays. Dose response analyses revealed that the percentages of labeled MDA-MB231 and MCF7 cells were increased with increasing concentrations of Nef-M1 peptide or protein (Figure 3). In contrast, there was no response by cell lines MDA-MB468 and HME, indicating a lack of CXCR4 expression (Figure 3). In comparison to untreated cells, there was more apoptosis in cells positive for CXCR4 expression when treated with either Nef-M1 or anti-CXCR4 Nef-M1 linked (αCXCR4/M1) monoclonal antibody (mab). For MDA-MB231 cells, the percent of labeled nuclei was 89.3% for Nef-M1 peptide treated cells and 12.0% for αCXCR4/M1-treated cells. MCF7 cells demonstrated 20.5% labeled nuclei after Nef-M1 peptide treatment and 5.7% after αCXCR4/M1 treatment (Figure 4). In all cases, more apoptosis occurred in MDA-MB231 and MCF7 cells treated with Nef-M1 peptide than in MDA-MB468 and HME cells treated in a similar fashion. Untreated MDA-MB231 and MCF7 cells had apoptotic responses similar to cells not expressing CXCR4.

### 3.3. Effects of Nef Protein and Peptides on Caspase-3 Activation in MDA-MB231 Cells

**Figure 5:**
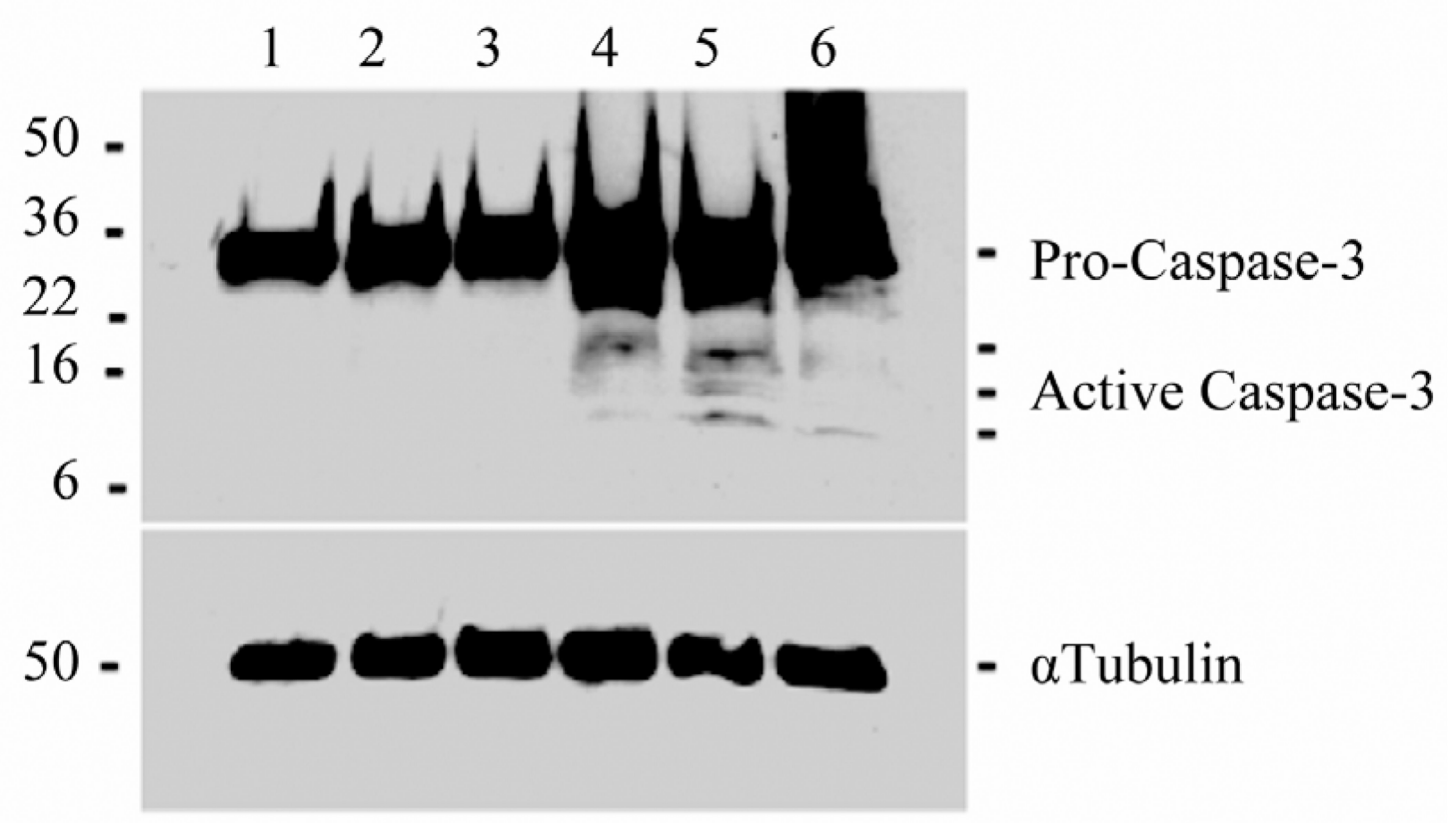
Effect of Nef peptides on caspase-3 activation in MDA-MB231 cells. Caspase-3 activation was determined by cleavage of the 32 kDa pro-caspase-3 protein into two smaller 17 kDa and 12 kDa caspase-3 proteins. Nef protein (lane 4) and Nef peptides [Nef 41 – 60 (lane 5), Nef-M1 (lane 6)] were efficient in inducing apoptosis, as determined after exposure for 48 hr, as determined by WBs, which show the activated caspase-3 in comparison to untreated (lane 1) cells and cells treated with Nef-sM1 (lane 2) or Nef 171 – 180 (lane 3).

The level of apoptosis in MDA-MB231 cells treated with Nef protein or peptides (Nef 171 – 180, Nef 41 – 60, Nef-M1, or Nef-sM1) was assessed by cleavage of the 32 kDa procaspase-3 protein into two smaller 17 kDa and 12 kDa proteins. As shown in Figure 5, Nef protein (lane 4) and Nef peptides [Nef 41 – 60 (lane 5), Nef-M1 (lane 6)], as determined by WB analysis after 48 hr of incubation, efficiently induced apoptosis in these cells by inducing activation of caspase-3. The high molecular weight procaspase-3 was found in untreated cells (lane 1) and in cells treated with Nef-sM1 (lane 2) or Nef 171 – 180 (lane 3).

### 3.4. Effect of Nef-M1 Peptide on Primary Tumor Growth and Metastasis of MDA-MB231 Cells

**Figure 6:**
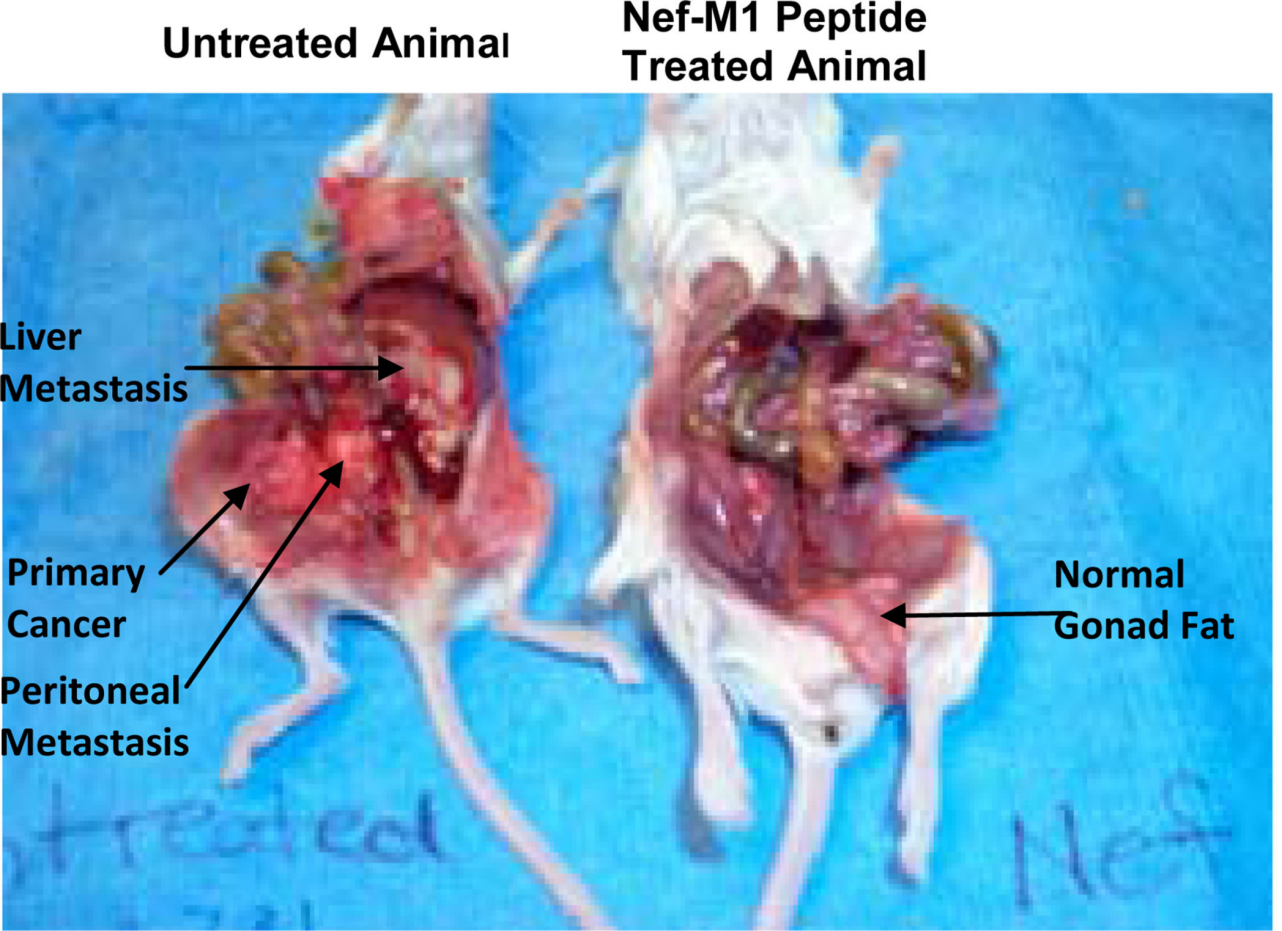
Effect of Nef-M1 on primary tumor growth and metastasis of MDA-MB231 tumors. The mice were either untreated (on the left) or treated with Nef-M1 (on the right), starting at the time of tumor implantation. The untreated mouse developed diffuse intraperitoneal metastases; in the treated mouse, the primary tumor did not propagate and no metastasis occurred. A normal appearing gonad fat pad, which had been implanted with cancer cells, is hanging from the pelvis next to the left leg. The gonad fat in the untreated mouse is incorporated in the tumor growing in the pelvis.

BC xenografts were derived from MDA-MB231cells, which had high expression of CXCR4. The effects of the Nef-M1 peptide on primary BC growth and metastasis were evaluated (Figure 6). SCID mice were treated with Nef-M1 peptide, starting at the time of tumor implantation. For these mice, there was no propagation of primary tumor and no metastasis. Volumetric measurements demonstrated that treated mice had tumors that were smaller than those in untreated mice (3.19 cm^3^ vs. 4.29 cm^3^) and smaller metastatic lesions (0.39 cm^3^ vs. 2.1 cm^3^) as compared to their untreated counterparts (data not shown). Thus, the untreated mice had larger primary tumor growth and more diffuse intraperitoneal metastasis. In addition, in treated mice, gonadal fat pads that had been implanted with cancer cells were clear of tumor tissue, but the gonadal fat pads in the untreated mice developed tumors.

## 4. Discussion

Previous results from our laboratory demonstrated, in SCID mice, inhibitory effects of the Nef-M1 peptide on the growth of primary CRC xenografts generated from fresh surgical specimens of human CRCs. The peptide has been found to be an inducer of apoptosis in CRC cells [11] and inhibitor of tumor growth and metastasis [10].

In the present report, to determine the effect of the Nef-M1 peptide as an inhibitor of BC progression, we focused on its impact on apoptosis of BC cells. We also used mouse models to determine its impact on primary tumor growth and metastasis. The Nef-M1 peptide was highly cytotoxic to a BC cell line expressing CXCR4, and the effect was relative to the presence of CXCR4 on the cell surface. Administered to mice with BCs, the peptide caused a reduction of primary tumor growth and inhibition of metastases.

Chemokine receptors, which belong to the family of G-protein-coupled receptors, are involved in regulation of the immune response, inflammation, leukocyte trafficking, and cytoskeletal rearrangement [13]. The chemokine receptor/ligand, CXCR4/SDF-1α is unique in that SDF-1α is the only known ligand for this receptor [14–18]. This is important because identifying a compound that interrupts this unique binding could have a profound impact as a therapeutic agent. The receptor/ligand pair induces strong chemotactic efficacy for leukocytes, and, in animals, is highly potent for chemoattraction [14–19]. Both CXCR4 and SDF-1α deficient mice display perinatal lethality owing to profound defects in embryonic development of the hematopoietic, cardiovascular, and nervous systems [14–18]. These phenotypic changes are mediated by the disrupted migration of embryonic progenitor cells into the appropriate microenvironment. These observations suggest that the SDF-1α/CXCR4 interaction is vital for the migration of non-hematopoietic, as well as hematopoietic cells. Studies with neutralizing antibodies to CXCR4 implicate this receptor in the homing and repopulation of human stem cells into the bone marrow of mice [20]. Furthermore, the CXCR4 receptor is a coreceptor for HIV-1 [21,22], and it is through this receptor that the virus causes cell death by apoptosis.

CXCR4 is highly expressed in solid human cancers, including breast [23], malignant melanoma [24], brain [25], anaplastic thyroid [26], non-small cell lung [27], pancreatic [28], ovarian [29], prostate [30], and colorectal [31]. In cells positive for myeloid differentiation antigen (Gr-1), expression of this chemokine receptor on the cell surface promotes metastasis by activating p38-activated protein kinase, suggesting that the receptor has a role in invasion and metastasis of cancer [32]. CXCR4 expression is higher in embryonic or dedifferentiated cells than in normal cells [33].

Tumor implantation, growth, and metastasis are dependent on neovascularization through angiogenesis [34, 35]. Over-expression of CXCR4 induces tumor metastasis through enhanced proliferation of cells caused by stimulating the MAP/Erk kinase pathway and through accelerating vascularization by activating vascular endothelial growth factor (VEGF) [36,37]. These mechanisms may be operative at primary sites as well as at distant sites throughout the life span of the tumor. In endothelial cells, the chemokine receptor/chemokine ligand, CXCR4/SDF-1α, is involved in growth factor-regulated signaling pathways. These pathways, linked to CXCR4, mediate steps in postnatal vascular remodeling and angiogenesis, which can lead to establishment and subsequent viability of tumors. Thus, targeting of CXCR4 by an appropriate therapeutic agent may be a means of controlling the aggressiveness of cancers.

Agents that specifically target the CXCR4 receptor have been developed [38,39]. By blocking the receptor from interacting with its natural ligand, inhibition of primary tumor growth and metastasis can be achieved. These CXCR4 antagonists, originally created to combat HIV-1, do not eliminate cells, but rather compete with the SDF-1α ligand. Apparently, the Nef-M1 peptide interacts with CXCR4 like other synthetic antagonists and inhibits primary tumor growth and metastasis. However, Nef-M1 also induces apoptosis in tumor cells [10,11]. Elevated levels of caspase-3 in surgical specimen xenografts that were treated with Nef-M1 peptide demonstrated the role of the peptide in induction of apoptosis. Caspases, which were essential for driving the apoptosis process, have been termed “executioner” proteins [40]. Consistent with previous reports, we have found the Nef-M1 peptide to be an efficient activator of caspase-3, a key molecule of the apoptotic process and a potential inhibitor of primary tumor growth and metastasis of BCs. Our findings suggest that the Nef-M1 peptide is a potential therapeutic agent that can be used to target tumor implantation, progression, and metastasis in BCs. We are in the process of a larger study with SCID mice on inhibition and metastasis of xenografts derived from surgical specimens of human BCs.

## References

[1] Siegel R, Naishadham D, Jemal A (2012). Cancer Statistics, 2012. CA: A Cancer Journal for Clinicians.

[2] Martin AM, Weber BL (2000). Genetic and Hormonal Risk Factors in Breast Cancer. Journal of the National Cancer Institute.

[3] Morrow PKH, Zambrana F, Esteva FJ (2009). Advances in systemic Therapy for HER2-Positive Metastatic Breast Cancer. Breast Cancer Research.

[4] Andre F, Xia W, Conforti R, Wei Y, Boulet T, Tomasic G, Spielmann M, Zoubir M, Berrada N, Arriagada R (2009). CXCR4 Expression in Early Breast Cancer and Risk of Distant Recurrence. The Oncologist.

[5] Burger JA, Kipps TJ (2006). CXCR4: A Key Receptor in the Crosstalk between Tumor Cells and Their Microenvironment. Blood.

[6] Ratajczak MZ, Zuba-Surma E, Kucia M, Reca R, Wojakowski W, Ratajczak J (2006). The Pleiotropic Effects of the SDF-1-CRXR4 Axis in Organogenesis, Regeneration and Tumorigenesis. Leukemia.

[7] Arya M, Patel HRH, Williamson M (2003). Chemokines: Key Players in Cancer. Current Medical Research and Opinion.

[8] Lee HJ, Kim SW, Kim HY, Li S, Yun HJ, Song KS, Kim S, Jo DY (2009). Chemokine Receptor CXCR4 Expression, Function, and Clinical Implications in Gastric Cancer. International Journal of Oncology.

[9] Muller A, Homey B, Soto H, Ge N, Catron D, Buchanan ME, McClanahan T, Murphy E, Yuan W, Wagner SN (2001). Involvement of Chemokine Receptors in Breast Cancer Metastasis. Nature.

[10] Harrington W, Bond V, Huang MB, Powell M, Lillard J, Manne U, Bumpers H (2009). HIV Nef-M1 Effects on Colorectal Cancer Growth in Tumor Induced Spleen and Hepatic Metastasis. Molecular and Cellular Pharmacology.

[11] Bumpers HL, Huang MB, Powell M, Grizzle WE, Lillard J, Okoli J, Bond VC (2005). Effects of HIV-1 Nef, a Cytotoxic Viral Protein, on the Growth of Primary Colorectal Cancer. Cancer Biology and Therapy.

[12] Huang MB, Jin LL, James CO, Khan M, Powell MD, Bond VC (2004). Characterization of Nef-CXCR4 Interactions Important for Apoptosis Induction. Journal of Virology.

[13] Rossi D, Zlotnik A (2000). The Biology of Chemokines and Their Receptors. Annual Review of Immunology.

[14] Moyer RA, Wendt MK, Johanesen PA, Turner JR, Dwinell MB (2007). Rho Activation Regulates CXCL12 Chemokine Stimulated Actin Rearrangement and Restitution in Model Intestinal Epithelia. Laboratory Investigation.

[15] Nagasawa T, Hirota S, Tachibana K, Takakura N, Nishikawa S, Kitamura Y, Yoshida N, Kikutani H, Kishimoto T (1996). Defects of B-Cell Lymphopoiesis and Bone-Marrow Myelopoiesis in Mice Lacking the CXC Chemokine PBSF/SDF-1. Nature.

[16] Zou YR, Kottmann AH, Kuroda M, Taniuchi I, Littman DR (1998). Function of the Chemokine Receptor CXCR4 in Haematopoiesis and in Cerebellar Development. Nature.

[17] Bleul CC, Fuhlbrigge RC, Casasnovas JM, Aiuti A, Springer TA (1996). A Highly Efficacious Lymphocyte Chemoattractant, Stromal Cell-Derived Factor 1 (SDF-1). The Journal of Experimental Medicine.

[18] Bleul CC, Farzan M, Choe H, Parolin C, Clark-Lewis I, Sodroski J, Springer TA (1996). The Lymphocyte Chemoattractant SDF-1 Is a Ligand for LESTR/Fusin and Blocks HIV-1 Entry. Nature.

[19] Murakami T, Nakajima T, Koyanagi Y, Tachibana K, Fujii N, Tamamura H, Yoshida N, Waki M, Matsumoto A, Yoshie O (1997). A Small Molecule CXCR4 Inhibitor that Blocks T Cell Line-Tropic HIV-1 Infection. The Journal of Experimental Medicine.

[20] Seeger FH, Rasper T, Koyanagi M, Fox H, Zeiher AM, Dimmeler S (2009). CXCR4 Expression Determines Functional Activity of Bone Marrow-Derived Mononuclear Cells for Therapeutic Neovascularization in Acute Ischemia. Arteriosclerosis, Thrombosis, and Vascular Biology.

[21] Manetti R, Cosmi L, Galli G, Annunziato F, Mazzetti M, Romagnani S, Maggi E (2000). Enhanced Expression of the CXCR4 Co-Receptor in HIV-1-Infected Individuals Correlates with the Emergence of Syncytia-Inducing Strains. Cytokines Cellular & Molecular Therapy.

[22] Berger EA, Murphy PM, Farber JM (1999). Chemokine Receptors as HIV-1 Coreceptors: Roles in Viral Entry, Tropism, and Disease. Annual Review of Immunology.

[23] Andre F, Xia W, Conforti R, Wei Y, Boulet T, Tomasic G, Spielmann M, Zoubir M, Berrada N, Arriagada R (2009). CXCR4 Expression in Early Breast Can cer and Risk of Distant Recurrence. The Oncologist.

[24] Scala S, Ottaiano A, Ascierto PA, Cavalli M, Simeone E, Giuliano P, Napolitano M, Franco R, Botti G, Castello G (2005). Expression of CXCR4 Predicts Poor Prognosis in Patients with Malignant Melanoma. Clinical Cancer Research.

[25] Stevenson CB, Ehtesham M, McMillan KM, Valadez JG, Edgeworth ML, Price RR, Abel TW, Mapara KY, Thompson RC (2008). CXCR4 Expression Is Elevated in Glioblastoma Multiforme and Correlates with an Increase in Intensity and Extent of Peritumoral T2-Weighted Magnetic Resonance Imaging Signal Abnormalities. Neurosurgery.

[26] Hwang JH, Hwang JH, Chung HK, Kim DW, Hwang ES, Suh JM, Kim H, You KH, Kwon OY, Ro HK (2003). CXC Chemokine Receptor 4 Expression and Function in Human Anaplastic Thyroid Cancer Cells. The Journal of Clinical Endocrinology and Metabolism.

[27] Spano JP, Andre F, Morat L, Sabatier L, Besse B, Combadiere C, Deterre P, Martin A, Azorin J, Valeyre D (2004). Chemokine Receptor CXCR4 and Early-Stage Non-Small Cell Lung Cancer: Pattern of Expression and Correlation with Outcome. Annals of Oncology.

[28] Wehler T, Wolfert F, Schimanski CC, Gockel I, Herr W, BielesterfeildI S, Seifert JK, Adwan H, Berger MR, Junginger T (2006). Strong Expression of Chemokine Receptor CXCR4 by Pancreatic Cancer Correlates with Advanced Disease. Oncology Reports.

[29] Jiang YP, Wu XH, Shi B, Wu WX, Yin GR (2006). Expression of Chemokine CXCL12 and Its Receptor CXCR4 in Human Epithelial Ovarian Cancer: An Independent Prognostic Factor for Tumor Progression. Gynecologic Oncology.

[30] Akashi T, Koizumi K, Tsuneyama K, Saiki I, Takano Y, Fuse H (2008). Chemokine Receptor CXCR4 Expression and Prognosis in Patients with Metastatic Prostate Cancer. Cancer Science.

[31] Kim J, Takeuchi H, Lam ST, Turner RR, Wang HJ, Kuo C, Foshag L, Bilchik AJ, Hoon DSB (2005). Chemokine Receptor CXCR4 Expression in Colorectal Cancer Patients Increases the Risk for Recurrence and for Poor Survival. Journal of Clinical Oncology.

[32] Hiratsukaa S, Dudaa DG, Huanga Y, Goela S, Sugiyamab T, Nagasawab T, Fukumuraa D, Jaina RK (2011). C-X-C Receptor Type 4 Promotes Metastasis by Activating p38 Mitogen-Activated Protein Kinase in Myeloid Differentiation Antigen (Gr-1)-Positive Cells. Proceedings of the National Academy of Sciences.

[33] Salvucci O, Yao L, Villalba S, Sajewicz A, Pittaluga S, Tosato G (2002). Regulation of Endothelial Cell Branching Morphogenesis by Endogenous Chemokine Stromal-Derived Factor-1. Blood.

[34] Folkman J (1990). What Is the Evidence that Tumors Are Angiogenesis Dependent?”. Journal of the National Cancer Institute.

[35] Fidler IJ, Singh RK, Yoneda J, Kumar R, Xu L, Dong Z, Bielenberg DR, McCarty M, Ellis LM (2000). Critical Determinants of Neoplastic Angiogenesis. Cancer Journal.

[36] Ferrara N, Gerber HP (1999). Vascular Endothelial Growth Factor: Molecular and Biological Aspects. Current Topics in Microbiology and Immunology.

[37] Ferrara N, Davis-Smyth T (1997). The Biology of Vascular Endothelial Growth Factor. Endocrine Reviews.

[38] Tamamura H, Xu Y, Hattori T, Zhang X, Arakaki R, Kanbara K, Omagari A, Otaka A, Ibuka T, Yamamoto N (1998). A Low-Molecular-Weight Inhibitor against the Chemokine Receptor CXCR4: A Strong Anti-HIV Peptide T140. Biochemical and Biophysical Research Communications.

[39] Liang Z, Wu T, Lou H, Yu X, Taichman RS, Lau SK, Nie S, Umbreit J, Shim H (2004). Inhibition of Breast Cancer Metastasis by Selective Synthetic Polypeptide against CXCR4. Cancer Research.

[40] Cohen GM (1997). Caspases: The Executioners of Apoptosis. Biochemical Journal.

